# A screening tool for non-specific low back pain with disability in office workers: a 1-year prospective cohort study

**DOI:** 10.1186/s12891-015-0768-y

**Published:** 2015-10-14

**Authors:** Prawit Janwantanakul, Rattaporn Sihawong, Ekalak Sitthipornvorakul, Arpalak Paksaichol

**Affiliations:** Department of Physical Therapy, Faculty of Allied Health Sciences, Chulalongkorn University, Bangkok, 10330 Thailand

## Abstract

**Background:**

Having a screening tool with reasonable predictive ability is essential in providing information about an individual’s risk of developing a disease, allowing an examination to be conducted with limited personnel and time, and selecting the relevant individuals for therapeutic research. This study aimed to produce a screening tool to identify office workers at risk of developing non-specific low back pain (LBP) with disability, and to evaluate the tool’s predictive power.

**Methods:**

At baseline, 615 healthy office workers filled out a self-administered questionnaire and underwent physical examination to gather potential risk factors. The incidence of LBP was collected every month thereafter. Disability level was assessed using the Roland-Morris Disability Questionnaire (RMDQ). The minimum RMDQ score for categorization as LBP was 3. Logistic regression was used to select significant factors to build a risk score. The coefficients from the logistic regression model were used to develop the components of a screening tool.

**Results:**

Over the 1-year follow-up, 8.8 % of participants reported incident LBP with disability. The screening tool for non-specific low back pain with disability in office workers comprised two items that contributed to the total score: previous history of LBP and psychological demand (assessed by the Job Content Questionnaire). The score range of the screening tool was 12 to 69. With a cut-off score of 53, the sensitivity was 65 % and the specificity was 68 %. The positive and negative predictive values were 16 and 95 %, respectively. The area under the receiver-operating characteristic curve was 0.76.

**Conclusions:**

A screening tool for non-specific low back pain with disability in office workers was developed and appears to have reasonable sensitivity, specificity, positive predictive values, and negative predictive values. Further validation and impact studies of the screening tool in a new population of office workers are suggested.

## Background

Over the past decade, musculoskeletal disorders have become a major disease causing disability and increased burden worldwide. This burden is likely to grow steadily because of rising rates with age, little change within individuals over time, and an ageing world population [1]. Studies have shown that between 34 and 51 % of office workers have experienced low back pain (LBP) in the preceding 12 months [2, 3], and 20 to 23 % of office workers report a new onset of LBP during a 1-year follow-up [4, 5]. A previous study also revealed that nearly a third of LBP patients had not completely recovered 12 months after the onset of LBP [6]. LBP is often the cause of significant physical and psychological health impairments. It also affects work performance and social responsibilities. As a result, LBP can be a great socioeconomic burden on patients and society [7]. In the United States, the total cost of LBP in 2006 exceeded 100 billion US dollars [8], whereas in the Netherlands the total cost of LBP in 2007 was estimated at 3.5 billion euros [9].

Having a screening tool to identify those likely to have LBP offers several benefits. First, such a tool provides evidence-based information about an individual’s risk of developing LBP, which will guide health professionals and individuals in joint decisions on further intervention. Identification of persons at risk would also mean the enhancement of resource allocation to those most in need and most likely to benefit from it. Without a screening tool, a large number of people would receive intervention, which is likely to compromise its effectiveness [10]. Second, a screening tool allows an examination to be conducted in primary health care and workplace settings where full clinical examinations are impractical due to limited personnel and time [11]. Lastly, a screening tool is beneficial for selecting the relevant individuals for therapeutic research. Researchers may use a validated screening tool to select healthy participants with an increased risk of developing a disease for a randomized controlled trial of a specific intervention to prevent a disease [10].

The etiology of musculoskeletal disorders is widely accepted to be of multifactorial origin, including individual, physical, and psychosocial factors. Different occupations are exposed to different working conditions and the nature of work influences the health of workers [12]. Predisposing factors for LBP are likely to be population-specific. In 2011, we proposed a screening tool to identify office workers likely to develop LBP [13]. To create the tool, we identified important biopsychosocial predictors, then assigned relative weights to each predictor and estimated the model’s predictive performance. A major weakness of this tool is that it was developed on the basis of cross-sectional data, which can only reveal an association between exposure and outcome, rather than a causal relationship. A prospective study, which permits the tracking of study participants’ activities, health status and exposures over time, is needed to determine the causal factors of a disease [14]. Also, those at risk of developing of LBP with disability can be identified and targeted as a prioritized group for receiving interventions. Thus, the purpose of this prospective cohort study is to use data from a 1-year prospective cohort study to develop a screening tool to assist health care providers in identifying office workers who are at risk of developing non-specific LBP.

## Methods

### Study population and procedures

The study recruited a convenience sample of office workers from nine large-scale enterprises in Thailand. The enterprises represented public transportation, infrastructure, energy, health care enterprises, a public university, a police department and three government ministries’ head offices. Office workers were defined as individuals working in an office environment with their main tasks involving use of a computer, reading, phoning, making presentations and participating in meetings. Other inclusion criteria were age between 18 and 55 years and working full-time. Exclusion criteria included reported musculoskeletal symptoms in the spine in the previous 3 months with pain intensity greater than 30 mm on a 100-mm visual analog scale; reported pregnancy or a plan to become pregnant in the next 12 months; history of trauma or accidents in the spinal region; and history of spinal, intra-abdominal or femoral surgery in the previous 12 months. Participants who had been diagnosed with a congenital anomaly of the spine, rheumatoid arthritis, infection of the spine and discs, ankylosing spondylitis, spondylolisthesis, spondylosis, tumor, systemic lupus erythematosus, or osteoporosis were also excluded from the study. Potential participants were screened for the study using a self-administered questionnaire.

**Fig. 1:**
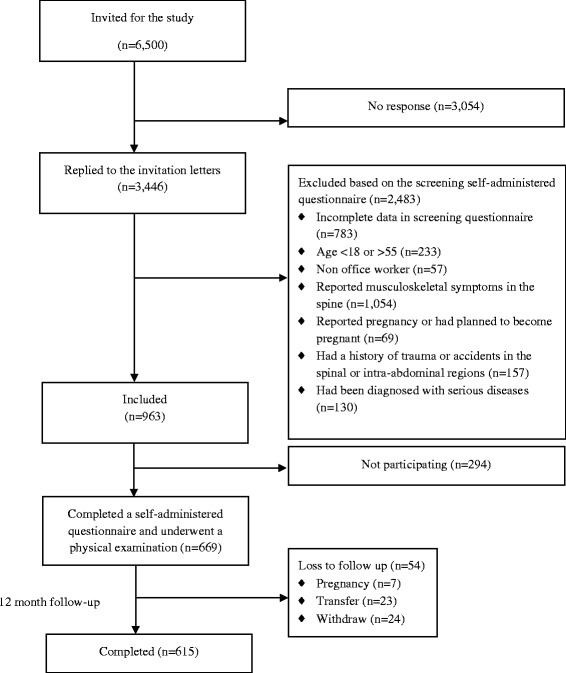
Flowchart of participants for the study

At baseline, participants completed the questionnaire and underwent physical examination by trained physical therapists according to a standardized protocol. Participants then received a diary in which they were instructed to record the incidence of LBP. The researcher returned to collect the diaries from participants every month over a 12-month period. Figure 1 shows the attrition data. All participants were given information about the study and signed a consent form before participating in the research. The study was approved by the Chulalongkorn University Human Ethics Committee.

### Questionnaire

The questionnaire gathered data on individual, work-related physical, and psychosocial factors. The selection of biopsychosocial factors for the study was based on two published reviews of the literature [15, 16]. In total, 49 biopsychosocial factors were identified. Individual factors included gender, age, marital status, educational level, frequency of regular exercise or sport, smoking habits, and number of driving hours a day. Work-related physical factors included current job position, number of working hours, years of working experience, frequency of using a computer, performing various activities during work, and rest breaks. The questionnaire also asked respondents to self-rate certain aspects of their work environment (ambient temperature, noise level, light intensity, and air circulation). Psychosocial factors were measured by the Job Content Questionnaire [17]. The questionnaire comprised 54 items in the following six areas: psychological demand (12 items), decision latitude (11 items), social support (8 items), physical demand (6 items), job security (5 items), and hazards at work (12 items). Each item had a four-point Likert-type response option ranging from 1, strongly disagree, to 4, strongly agree.

### Physical examination

Body weight and height were measured by a digital scale and a wall-mounted stadiometer, respectively. Waist circumference was measured midway between the lower rib margin and the superior border of the iliac crest using a tape measure [18]. Trunk extension flexibility was assessed by the modified Schober test [19]. Erector spinae and multifidus muscle endurance was assessed by the Biering-Sorensen test [20].

Before the primary data collection, test-retest reliability of data from the questionnaire and physical examination was assessed in a subsample of 20 participants. Each participant was tested on two occasions separated by a period of 7 days for the questionnaire and 10 min for the physical examination.

### Outcome measures

The incidence of non-specific LBP was collected using a diary. The locus of the pain was defined according to the picture of the body from the standardized Nordic questionnaire [21]. Non-specific LBP is LBP (with or without radiation) without any specific systematic disease being detected as the underlying cause of the complaints [22]. Participants answered the yes/no question “Have you experienced any LBP lasting > 24 h during the past month?” If they answered “Yes”, follow-up questions about pain intensity measured by a visual analogue scale (VAS) and the presence of weakness or numbness in the lower limbs were asked. Those who reported an incidence of LBP were asked about their disability level as measured by the Roland-Morris Disability Questionnaire (RMDQ-24) [23]. The RMDQ contains 24 yes/no items and patients are asked whether the statements apply to them that day (the last 24 h). The RMDQ score is calculated by adding up the number of “yes” items, ranging from 0 to 24, with higher scores indicating more severe disability.

In this study, participants were identified as cases if, in any month during the follow-up, they answered ‘Yes’ to the first question, reported pain intensity greater than 30 mm on a 100-mm visual analog scale, had no weakness or numbness in the lower limbs, and had an RMDQ score of at least 3. A previous study indicated that a cut-off of 2 on the RMDQ most accurately classifies participants as recovered from LBP [24]. Participants were followed until they become symptomatic, withdrew from the study, or completed the 12-month follow up.

### Statistical analysis

For the reliability study, the intraclass correlation coefficient (ICC) was calculated for continuous data and the Phi coefficient for nominal data. The ICC (3,1) was calculated for the questionnaire and physical examination outcomes.

Characteristics of participants were described using means or proportions. The percentages of missing data for the individual, work-related physical, and psychosocial factor categories were in the range of 0.3 %–2.8 %. To retain the statistical power of the database, missing data were handled using the ‘hot-deck imputation’ procedure: A case in which information was missing was replaced by the value from a randomly selected case from the total sample of the study. This procedure was conducted repeatedly for each missing value until the dataset was complete [25].

The 1-year incidence rate of non-specific LBP with disability was calculated as the proportion of new cases, defined as not having had LBP at baseline but reporting it at a disability level that met the criterion (RMDQ ≥ 3) during the follow-up.

To develop a risk score to predict incidents of non-specific LBP with disability in office workers, a series of statistical analyses was conducted. The associations between each factor and LBP were evaluated using univariate logistic regression analysis. Any factors with a *p*-value ≤ 0.1 were eligible for addition into multivariate analysis. Multivariate logistic regression analysis with backward stepwise selection was then performed to determine the optimal combination of biopsychosocial factors needed to predict incident LBP with disability. Statistical significance was set at the 5 % level.

Before univariate logistic regression analysis was conducted, collinearity between the different predictor variables was checked using the Variance Inflation Factors (VIF) and Tolerance. Collinearity was assumed to be present if VIF was higher than 10 and Tolerance was lower than 0.1 [26]. If collinearity was present, the risk factor with the highest correlation with the outcome was used for the multivariable analysis. The ‘explained variance’ of each of the multivariable logistic regression models was calculated by means of Nagelkerke’s R^2^ and the goodness of fit by means of the Hosmer and Lemeshow goodness-of-fit test [27].

A simplified scoring system was developed on the basis of coefficient results. A score was assigned to each variable based on the magnitude of the β coefficient. A total score for the risk of developing LBP was calculated as the sum of each variable. A receiver-operating characteristic (ROC) curve and the area under the curve (AUC) were produced to evaluate the discriminatory ability of the risk score. Sensitivity, specificity, positive predictive value (PPV) and negative predictive value (NPV) for several cut-off scores were calculated. The cut-off score that gave the maximum sum of sensitivity and specificity was taken as an optimum. All statistical analyses were performed using SPSS for Windows Version 17.0 (SPSS Inc., Chicago, IL).

## Results

The test-retest reliability results demonstrated fair (0.53) to excellent (1.00) reliability for the questionnaire data and good (0.72) to excellent (1.00) reliability for the physical examination data.

A total of 669 workers agreed to participate in the physical examination (Fig. 1). Six hundred and fifteen participants were followed for 1 year, and 54 (8.1 %) participants were lost during the follow-up period due to pregnancy (*n* = 7), job transfer (*n* = 23), and withdrawal (*n* = 24). Table 1 presents the baseline characteristics of the study population. Over the 1-year follow-up, the incidence of non-specific LBP with disability (RMDQ ≥ 3) in the sample population was 8.8 % (54/615) with mean (SD) VAS and RMDQ scores of 47 (17) and 3.4 (2.7), respectively.

**Table 1:** Characteristics of study population (*n* = 669)

Characteristics	N (%)	Mean ± SD
*Demographic characteristics*		
Gender		
Male	168 (25.1)	
Female	501 (74.9)	
Age (years)		35.7 ± 8.3
20–29	189 (28.2)	
30–39	265 (39.6)	
40–49	161 (24.1)	
≥ 50	54 (8.1)	
Education		
Lower than Bachelor’s degree	83 (12.4)	
Bachelor’s degree	478 (71.5)	
Higher than Bachelor’s degree	108 (16.1)	
Exercise frequency in the past 12 months		
Never	158 (23.6)	
Occasionally	429 (64.1)	
Regularly	72 (10.8)	
Not sure	10 (1.5)	
History of LBP		
Yes	525 (78.5)	
No	144 (21.5)	
*Occupational-related characteristics*		
Duration of employment (years)		10.7 ± 8.5
Working hours per day (hours per day)		7.9 ± 1.1
Working days per week (days per week)		5.0 ± 0.5
Frequent rest break		
Yes	543 (81.2)	
No	126 (18.8)	
*Psychosocial characteristics*		
Job control		35.0 ± 4.4
Psychological demand		32.3 ± 4.4
Physical demand		13.4 ± 2.6
Job security		16.4 ± 1.9
Social support		30.6 ± 4.6
Hazards at work		16.7 ± 3.6
*Physical characteristics*		
Weight (kg)		60.3 ± 13.9
Height (cm)		160.1 ± 7.3
Body mass index (kg/m^2^)	70 (10.5)	
< 18.5 kg/m^2^	409 (61.1)	
18.5–24.9 kg/m^2^	126 (18.8)	
25–29.9 kg/m^2^	64 (9.6)	
30 kg/m^2^		
Waist circumference		77.7 ± 11.3
Trunk extension flexibility (cm)		13.0 ± 0.8
Erector spinae and multifidus endurance (s)		71.7 ± 38.0

The effect of missing data on the findings of the present study was investigated by comparing the results before and after performing the ‘hot-deck imputation’ procedure, and no difference was found. Therefore, the results after the ‘hot-deck imputation’ procedure are presented here.

In the univariate logistic regression analysis, variables showing *p*-value < 0.1 were body weight, history of LBP, physical demands, and psychological demands. Multivariate logistic regression analysis revealed a significant association between onset LBP with disability and history of LBP and psychological demand (Table 2). Nagelkerke’s R^2^ was 0.062 and the Hosmer-Lemeshow goodness-of-fit test was not significant (*χ*^2^ = 13.834, *p* = 0.086). To develop a screening tool for non-specific LBP with disability in office workers, scores were assigned to each variable (Table 3). A screening tool comprised 2 items that contributed to the total score: history of LBP and psychological demand (assessed by the Job Content Questionnaire). Each item was unequal in weight. The history of LBP question was worth 21 points for a “yes” answer. For psychological demand, respondents were required to answer 12 questions in the psychological demand section of the Job Content Questionnaire. Each question had a four-point Likert response scale ranging from 1, strongly disagree, to 4, strongly agree. Thus, scores for the psychological demand question ranged from 12-48. The total score for an individual could range from 12 to 69, with a higher score indicating higher risk of developing non-specific LBP with disability. The optimal cut-off score was 53 (sensitivity = 64.8 %; specificity = 67.9 %; PPV = 16.3 %; NPV = 95.2 %). The AUC was 0.76 (95 % CI 0.68-0.83).

**Table 2:** Incidence and adjusted odds ratio (OR_adj_) with 95 % confidence interval (95%CI) of disabling, non-specific low back pain with respect to factors in the final modelling (*n* = 615)

Factors	N	Incidence (%)	OR_adj_	95%CI	*P* value
History of low back pain					
Yes	484	51 (10.54)	5.31	1.63–17.36	0.006
No	131	3 (2.29)	1.00		
Psychological demand (assessed by the Job Content Questionnaire)			1.08	1.02–1.15	0.014

^a^Factors included in the statistical modelling were body weight, history of LBP, physical demands, and psychological demands

^b^All ORs associated with particular factors were adjusted for the effect of all other factors that were in the model

**Table 3:** A screening tool for non-specific low back pain with disability in office workers

Factors	β Coefficient	Risk score^a^
History of low back pain		
Yes	1.67	21
No		0
Psychological demand		
Scores derived from the psychological demand section of the Job Content Questionnaire^b^ (Score ranges from 12 to 48)	0.078	X
Total score	21 + X	

^a^β Coefficient of psychological demand was assigned a score of 1 and then the other β Coefficient was divided by 0.078 and rounded off to the nearest integer

^b^The Job Content Questionnaire comprises 54 items that address the following six areas: psychological demand (12 items), decision latitude (11 items), social support (8 items), physical demand (6 items), job security (5 items), and hazards at work (12 items). Each item has a four-point Likert response scale ranging from 1, strongly disagree, to 4, strongly agree

## Discussion

The main purpose of this study was to create a screening tool to identify office workers at risk of developing non-specific LBP with disability. Various individual, work-related physical, and psychosocial factors as well as outcomes from a physical examination conducted by trained physical therapists were included in the analysis. The results demonstrated that individual (history of LBP) and psychological (job demand) factors were two predictors of non-specific LBP with disability in our sample. Although history of LBP is non-modifiable risk factor, the screening tool is a potentially useful tool for helping clinicians identify office workers at risk of developing non-specific LBP with disability. Identification of persons at risk would mean the enhancement of resource allocation to those most in need and most likely to benefit from preventive intervention. Without a screening tool, a large number of people who did not need the intervention would likely receive it, which is likely to compromise its effectiveness [10, 28]. The developed screening tool is relatively easy to administer and can be carried out within a short space of time (approximately 5 min) because it requires a respondent to answer just 13 questions: one question regarding history of LBP and 12 questions regarding psychological demand. Therefore, it is suitable for primary health care and workplace settings, where full clinical examinations are impractical due to limited personnel and time.

This study found the annual incidence of non-specific LBP with disability in office workers to be 8.8 %. The sample had moderate pain intensity levels but low disability levels. Rantonen et al. [29] reported that the average RMDQ score (RMDQ-18) in workers with mild LBP (i.e., LBP with VAS 10–34 mm) was 3. Hill et al. [30] found the average VAS and RMDQ-24 scores in adults with back pain consultations (with or without radiculopathy) at general practices to be 5.2 mm and 9.7 mm, respectively. In the current study, office workers who reported incident LBP still continued to work. Workers who keep working would be expected to have low disability because it would be difficult for them to remain productive with high disability levels [31].

Predictors in the screening tool reported in the present study differed from those that have been reported previously [13] and that were developed based on data from a cross-sectional study, including previous history of working as an office worker, years of work experience, continuous standing for > 2 h a day, frequency of forward bending during the work day, chair having lumbar support, and backache index outcome. A recent systematic review on prospective cohort studies found strong evidence for history of LBP and limited evidence for the combination of postural risk factors and job strain (for females only) as predictors of the onset of non-specific LBP in office workers [16]. In their systematic review of longitudinal studies, da Costa and Vieira [32] identified several risk factors for work-related musculoskeletal disorders in the low back, including negative affectivity, low level of job control, high psychological demands, and high work dissatisfaction.

Selection of an optimal cut-off point largely depends on the purpose of using the risk score and requires knowledge of the sensitivity, specificity, PPV, and NPV. In the present study, a cut-off score of ≥ 53 provided the maximum sum of sensitivity and specificity. The sensitivity is 65 %; consequently, the false-negative rate is 35 %. A high false-negative would result in greater medical expenses and disability for a disease later on because those high-risk workers would be missed. With a cut-off score of ≥ 53, the specificity is 68 %, and the false-positive rate is 32 %. Because these low-risk office workers may not have received any benefits from any preventive intervention given to them, a high false-positive rate would cost money and time. One needs to consider the expected consequences of missing a person at risk (false-negative) as opposed to including a person in an intervention, although they are not at risk (false-positive). For example, with limited resources, one may want to increase the likelihood of including those who are truly at risk of developing LBP with disability. Therefore, a screening tool with high specificity would be preferable to one with high sensitivity. In contrast, to significantly reduce the number of office workers with disabling LBP, one may prefer a screening tool with high sensitivity to one with high specificity to ensure that as many of those high-risk workers will receive preventive intervention as possible.

In practice, predictive values may be more useful than sensitivity and specificity rates for applying the screening tool in clinical decision making, because predictive values indicate the probability that the result is correct [33]. Our results show that the predictive value of the cut-off of ≥ 53 was low for the PPV and high for the NPV. The PPV was 16 %, indicating that 16 % of office workers with a score of ≥ 53 are actually at risk of developing LBP with disability. The NPV was 95 %, meaning that 95 % of office workers with a score of < 53 were not at risk for developing LBP with disability. Based on the findings, the screening tool developed in the current study seems to be suitable for ruling out healthy office workers with a low risk of developing LBP with disability, rather than for ruling in those with a high risk of developing LBP with disability. Although the PPV and NPV provide useful information for interpreting the screening tool, they are highly dependent on the prevalence of the condition of interest in the sample: the PPV will be lower and the NPV will be higher in samples with a low prevalence of the condition [33].

A major strength of this study is its prospective design, which allows for the identification of cause-effect relationships and the evaluation of a broad range of biopsychosocial factors for their contribution to non-specific LBP with disability in office workers. In addition, a large sample was successfully followed up for 1 year (92 %), which enabled robust results for determining the model’s goodness of fit. However, at least three main limitations are noteworthy. First, this was a development study of a prognostic model. The predictive performance of the screening tool was tested on the same sample in which the screening tool was developed. The model is likely to perform better in the development sample than in an independent sample. In addition, the results with the screening tool may be specific to this population; therefore, any extrapolation of the results should be made with caution. Further research to validate or test the screening tool’s predictive performance in a different population of office workers is suggested. Also, impact studies to quantify whether use of the screening tool in daily practice improves decision-making and patient outcome are recommended [10]. Second, in this study, participants were identified as cases if they reported pain intensity greater than 30 mm on a 100-mm VAS, had no weakness or numbness in the lower limbs, and had an RMDQ score of at least 3. Different screening tools may emerge with different definitions of symptomatic cases, such as changing the cut-off point of pain intensity to less than 30 mm on a 100-mm VAS or increasing the cut-off point of the RMDQ score for disability. Third, there were a relatively small number of cases (i.e., those reporting incident LBP with disability), resulting in poor fit of the multivariable logistic regression model to the data (Nagelkerke’s R^2^ = 0.062). As a result, the cut-off score of the screening tool that gave moderate predictive power (i.e., 65 % sensitivity and 68 % specificity) was relatively high (i.e., ≥ 53 out of 69). A prospective study is needed in which relatively high numbers of cases are recruited and other relevant biopsychosocial characteristics are evaluated, to improve the accuracy of the regression model and, consequently, the predictive performance of the screening tool.

## Conclusions

The screening tool developed in this study is easy to use and can be carried out within a short space of time. Within the limitations of the study, the screening tool is valuable in two complementary ways: it can help preserve resources and reduce hardship to workers by assisting in early identification of and intervention with those at high risk of developing non-specific LBP-related disability; and it can also help to rule out workers at low risk for such an outcome. However, further validation and impact studies of the screening tool in a new population of office workers are suggested.

## Abbreviations

LBP: Low back pain
RMDQ: Roland-Morris Disability Questionnaire
VAS: Visual analogue scale
ICC: Intraclass correlation coefficient
VIF: Variance inflation factors
ROC: Receiver-operating characteristic
AUC: Area under the (ROC) curve
PPV: Positive predictive value
NPV: Negative predictive value
